# Purple Yampee Derivatives and Byproduct Characterization for Food Applications

**DOI:** 10.3390/foods13244148

**Published:** 2024-12-21

**Authors:** Sandra V. Medina-López, Cristian Molina García, Maria Cristina Lizarazo-Aparicio, Maria Soledad Hernández-Gómez, Juan Pablo Fernández-Trujillo

**Affiliations:** 1 Instituto de Ciencia y Tecnología de Alimentos (ICTA), Universidad Nacional de Colombia, Bogotá 111321, Colombia; svmedinal@unal.edu.co (S.V.M.-L.); mlizarazoa@unal.edu.co (M.C.L.-A.); mshernandez@unal.edu.co (M.S.H.-G.); 2 Departamento de Ingeniería Agronómica, Universidad Politécnica de Cartagena, 30203 Cartagena, Spain; 3 Servicio de Apoyo a la Investigación Tecnológica (SAIT), Universidad Politécnica de Cartagena, 30202 Cartagena, Spain; cristian.molina@upct.es; 4 Instituto Amazónico de Investigaciones Científicas (SINCHI), Bogotá 110311, Colombia

**Keywords:** circular economy, byproducts valorization, bioeconomy, natural ingredient, tuber, phenolics, anthocyanins, flours, HPLC-DAD-FLD, HPLC-ESI-QTOF-MS, HPAEC–PAD

## Abstract

This study assessed the technological potential and bioactive compounds present in purple yampee (*Dioscorea trifida* L.f.) lyophilized powder, peeled and whole flour, as well as the tuber peel, starch residual fiber, and wastewater mucilage. Although most values approached neutrality, flour showed a lower pH and high density, while greater acidity was observed in the mucilage. Color differed statistically and perceptibly between all samples, with similar values of °hue to purple flours from other sources, and the maximum chroma was found in lyophilized pulp and lightness in fiber. Average moisture levels around 7.2% and water activity levels of 0.303 (0.194 for whole flour) in fractions suggested favorable storability, while the interaction of the powders with water was similar to other root and tuber powdered derivatives. Yampee periderm had the highest swelling power, oil absorption capacity, water holding capacity, and absorption index and capacity. Mucilage had a higher solubility index and outstanding emulsion activity, greater than 90%. Twelve anthocyanins, with new reports of petunidin derivatives for the species, and more than 30 phytochemicals were identified through advanced liquid chromatography techniques. The greatest amounts of pinitol and myo-inositol were found in the mucilage, and sucrose, glucose, and fructose prevailed in the other powders. Successfully characterized yampee fractions showed high potential as functional food ingredients.

## 1. Introduction

International organizations have recognized, for more than two decades, that our food system is facing great pressure from many aspects, such as a growing population, which may demand a 70% increase in agricultural production for 2050 [1]. With a system where many smallholder farmers still rely on rain to irrigate their crops, now common phenomena of droughts and floods have put pressure on the crops’ survival as have algid cold and intense heat conditions that could affect produce yield and nutritional value. In fact, extreme weather events and temperature and precipitation pattern shifts that characterize climate change have been linked to a significant threat to agricultural systems and, thus, food security all over the globe according to multiple authors that defend local biodiversity as a naturally resilient instrument [2,3,4,5]. Among the solutions, besides the increase in production, food waste reduction is one of the first to stand out, considering how the edible portion—after removing inedible peels, seeds, or other structures—in many fruits and vegetables could serve several purposes. Instead of following a circular system that can emulate natural processes to promote resource efficiency in a bioeconomy model through secondary industries, the present generation of waste and byproducts along the food supply brings forth natural resource jeopardy, energy surplus, and a major contribution to environmental problems as agriculture is currently responsible for approximately a quarter of the global greenhouse emissions, leaving the economic and social impacts behind [6].

Second to market leaders’ cereals, roots and tubers are recognized as important carbohydrate sources with the advantage of readily adapting to diverse farming, environmental, and soil conditions with low inputs, yielding a wide array of bioactive compounds with proven hormonal, antioxidant, anticancer, anti-ulcerative, hypoglycemic, hypocholesterolemic, immunomodulatory, antimicrobial, and anti-obesity activities [7]. Long-term preservation of many of these roots and tubers commonly includes drying them into flour or extracting starch to increase the foods availability, particularly with crops of peak-harvest times. Natural effects of processing or tuber storage may decrease bioactive compounds, including complex carbohydrates that break into simple sugars as dormancy eases off or maturity progresses [8]. Such molecular rearrangement could change the availability of bioactive-functional carbohydrates and have sensory or technological consequences as yield decreases in the extraction of ingredients like starch or nonconventional hydrocolloids, whose processing waste may also rise [9]. As one of the world’s most important ingredients in food and other industries, starch production is known to originate large amounts of wastewater with a high chemical oxygen demand, biological oxygen demand, and suspended solids, which may lead to river hypoxia and, ultimately, threats to human living environments unless they are treated through adsorption, air flotation separation, biological treatments, reactors, flocculation, and precipitation or sedimentation methods [10]. Some of these methods could be expensive, impractical, or time- and energy-consuming though, so depending on the scale of the treating industry, strategies to prevent the waste by taking advantage of the byproducts may be more effective. It is estimated that food loss and waste add up to one-third of the food we seed, and waste only generates around 8–10% of global greenhouse emissions, a great resource misuse considering evidence has shown that investing in food loss and waste reduction may return multiplied by at least 14 times the original value [11].

Most food byproducts still hold a considerable amount of macro and micronutrients with both functional and nutritional potential useful for the development of foodstuff or food additives, which could entail a strong tool for minimizing malnutrition and hunger, diversifying productive chains that can generate social benefits with new job opportunities, and improving food security in developing countries [12]. In the case of aroids and yams, waste has been valorized through fuel production—in biogas and bioethanol forms, in biopolymers, as energy storage materials, for wastewater treatment, and as functional food additives [13]. Some whole tuber and root (R&T) flours display superior performance in 3D printing, easing the extrusion and showing a higher accuracy than their starch counterparts [14]. Contrary to refined flours, whole R&T flours show an enhanced nutritional profile as whole taro root flour, noted for its high fiber content, vitamins, and phytochemicals attractive for functional foods [15], while technological advantages may include cold paste stability, chewiness, and ductility, as in whole powdered lotus [16]. Despite this, most underground storage organs are usually peeled to improve appearance, flavor, and texture by removing fibrous, bitter substances or structures that may also contain antinutritional or toxic compounds, resulting in a first biomass waste. Yam peels have proven to modulate intestinal microflora and enhance the gut defense barrier, therefore improving the immunity of common carp [17] and being a good substrate for citric acid production [18].

Numerous local resources are yet to be explored for their industrial and bioactive potential, and South American purple yampee *Dioscorea trifida* L.f. is a pigmented underutilized tuber that has stood out for its atypical starch, anthocyanin content, and potential use for the industry with good performance in antioxidant-rich baked goods [19,20,21,22]. Carbonized tuber fragments suggest yam consumption in South America dating from at least 6000 years before the present according to southern Brazil evidence, with *D. trifida* crops all over the country as one of the most cultivated yams, known by farmers to be the ones with the “tastiest” tubers in addition to other health benefits [23]. Few recent studies exist on edible uses of yampee, a tuber of significant socioeconomic relevance, which has hinted to be rich in bioactive phytochemicals and, particularly in purple varieties, it is considered useful for quick-cooking foods in flour or starch forms, as a sustainable choice to ease commercialization [24]. Among many innovative approaches to promote sustainability in the food industry, “retro” perspectives in supply chains are advised [25] as traditional ways to preserve tubers by drying them into flours. We proposed, additionally to increase the efficiency of resource use with “waste handling” for this study, as an alternative for a comprehensive use of this tuber, yet to be explored with derivatives that may be of great value. As the species starch characteristics, performance, extraction, and modification methods -with corresponding effects- have been widely described [21,25,26,27,28,29], this study focused on the evaluation of the technological and functional potential of the starch’s byproducts and other derivates of the tuber as a way to promote its use in the food industry.

## 2. Materials and Methods

### 2.1. Biological Material and Chemical Reagents

Purple yampee (*D. trifida*) tubers were harvested in the Montes de Maria region from the North Colombian Caribbean, transported overland to a distributor, and sent, in a two hours flight to Bogotá, towards the Food Science and Technology Institute (ICTA) of the Universidad Nacional de Colombia for further processing and analysis. Myo-inositol chemical standard was acquired from Honeywell’s Research Chemicals (Morris plains, NJ, USA) acetonitrile (99.9% HPLC and LC/MS grade), methanol (99% HPLC grade), formic acid (LC/MS grade), standards of alanine, arginine, aspartate, glycine, histidine, isoleucine, leucine, lysine, serine, threonine, tyrosine, and valine from Agilent Technology™ (Santa Clara, CA, USA); peonidin 3-O-glucoside, D-(+)-Glucose, D-(-)-Fructose, Sucrose and D-Pinitol standards were purchased from Sigma-Aldrich^®^ (Burlington, MA, USA).

Upon receipt, basic conditioning operations were executed by weighing, washing, and disinfecting yampee tubers prior to manual peeling to obtain the first byproduct (periderm peel), rinse, and cut into 3 mm slices. Manually peeled periderm, sliced flesh, and whole (unpeeled) sliced tubers were dried at 60 °C for 24 h, grinding with a Robot coupe, Blixer 2^®^ (Ridgeland, MS, USA), and vacuum-packaged, thus obtaining Yampee Periderm Powder (YPP), Yampee Peeled Flour (YPF) and Whole Tuber Powder (WTP), the first derivatives, for storage in darkness at room temperature (20 ± 1.5 °C) until analysis.

Methods described by [21,29,30] were followed to extract the starch, recovering the resulting solid and liquid byproducts. In the process, peeled tubers were processed for 5 min in an industrial blender, sifted (reserving the resulting fiber to dry and grind, obtaining the Yampee Starch Fiber, YSF), and the slurry was centrifuged for 5 min at 3000× *g* at 4° C. Liquid supernatant was reserved, and the precipitate was washed three times by resuspending with distilled water (1:1). All fractions of supernatant were gathered to obtain the Yampee Starch Mucilage (YSM) fraction, frozen at −40 °C before freeze-drying with a maximum temperature of 40 °C and a pressure of 0.05 mbar [31] in a Labconco Freezone 12 (Kansas City, MO, USA) lyophilizer. Washed precipitates from the slurry were dried in a convection oven at 45 °C for 24 h, after which the starch was ground, sifted (mesh 60), and vacuum-packed for other studies. Because of the interest in the bioactive potential of the purple flesh inside the tuber, peeled tubers were blended and freeze-dried as the mucilage, to achieve the Lyophilized Yampee Pulp (LYP) fraction.

### 2.2. Technological Performance of the Fractions

Powdered yampee derivatives and byproduct technological properties of relevance for the industry, such as color, water activity (Aw), moisture, total titratable acidity (TTA), pH, water absorption capacity (WAC), oil absorption capacity (OAC), swelling power (SP), water solubility index (WSI), water absorption index (WAI), loose bulk density (LBD), packed bulk density (PBD), and apparent density (AD) from the LBD/PBD ratio, were assessed as described in [32]. The emulsion activity (EA), and water holding capacity (WHC) were measured as mentioned in [33]. Modifications included the use of Color Quest^®^ XE (HunterLab, Reston, VA, USA) with D65/10° illuminant/observer to measure color and the thermogravimetric method using a drying stove to measure moisture [34].

### 2.3. Bioactives Assessment

The detection and identification of phytochemicals of interest in *D. trifida* by HPLC-ESI-QTOF-MS were achieved following the methods in [35], with extraction adjusted by adding 5 mL of MetOH/H_2_O (50:50 *v*/*v*, with 0.1% formic acid) to 500 mg of powdered sample, vortexing (Heidolph^®^ Reax top, Schwabach, Germany) and sonicating (Ultrasonic Unit FB 15061 Fisher Scientific, Waltham, MA, USA) for 15 min before centrifuging, collecting supernatant, and re-extracting precipitate twice with 2.5 mL of solvent. The three supernatant fractions were gathered, filtered (0.20 μm), and cold stored (4 ± 0.5 °C) until analysis on the same day. The Agilent 1290 Infinity II LC (Santa Clara, CA, USA) equipped with a G7104A quaternary pump, G7167B Standard Autosampler, G7116B column heater, and coupled to a model G6546A hybrid quadrupole-time-of-flight mass spectrometer (Q-TOF) with a dual AJS electrospray ionization source (ESI) was used for this purpose. The separation of compounds was achieved on a reverse-phase Agilent^®^ column ZORBAX Eclipse XDB-C18 (4.6 × 150 mm; 5 μm).

Amino acid extraction was carried out by weighing 500 mg of the samples, adding 5 mL of 0.1 M HCl, homogenizing by vortex for sixty seconds before centrifuging at 3220× *g* and 20 °C for five minutes (A-4-62 rotor, Eppendorf^®^ 5810 R Centrifuge, Hamburg, Germany) before depuration (0.22 μm filter) and OPA/FMOC sample derivatization. The aforementioned HPLC equipment was used for determination, with the Agilent 1260 Infinity II Fluorescence Detector Spectra module, and the Zorbax Eclipse Plus C18 column (2.1 × 100 mm, 1.8 μm) following the methodology in [2]. Quantification of the compounds was performed using chemical standards for calibration curves with R^2^ ≥ 0.99.

Anthocyanin extraction was performed with a few modifications from [36]. A 500 mg sample of powdered tubers was suspended in 10 mL HPLC grade water with 0.3% formic acid, homogenized for 5 min by vortex, and centrifuged in 16 × 95 PP 400,900 tubes (Deltalab, Barcelona, Spain) at 3220× *g* and 4 °C for 10 min (A-4-62 rotor, Eppendorf^®^ 5810 R Centrifuge, Hamburg, Germany) before purification (0.20 μm filter), and then stored in darkness until measuring. Anthocyanin analysis was performed using an Agilent 1290 Infinity II LC System (Santa Clara, CA, USA) equipped with a G7104A quaternary pump, G7167B Standard Autosampler, G7116B column heater, and coupled to a model 1260 Diode Array Detector (DAD WR G7115A) and Agilent^®^ (Santa Clara, CA, USA) ZORBAX Eclipse XDB-C18 reverse phase column (4.6 mm × 150 mm × 5 μm), following the [22] method with an addition of 7 min after-time coming to the initial concentration of B phase to prevent crossover. Main anthocyanins were identified using Agilent® (Santa Clara, CA, USA) METLIN master accurate mass compound database and accurate mass MS/MS spectral library (version 8.0) and were quantified interpolating into the peonidin-3-glucoside standard calibration curve for identical or structurally related compounds (equivalents), as described by [35].

Sugar extracts for high-performance anion-exchange chromatography coupled with pulsed amperometric detection (HPAEC–PAD) were prepared by modifying the [37] procedure by suspending 300 mg of the sample in 10 mL of ultra-pure water and filtering through a 0.20 μm polyamide/nylon membrane (Chromafil^®^ Xtra, Macherey-Nagel, Düren, Germany). Samples were injected with a Metrohm™ (Herisau, Switzerland) 818 IC pump in 10 μL aliquots at a 0.5 mLmin^−1^ flow into a Metrohm^®^ 871 Advanced Bioscan Pulsed Amperometric Detector, with an isocratic flow of 300 mM NaOH and 1 mM NaOAc eluent for 15 min, with an oven temperature of 32 °C before detection on a Metrohm^®^ 945 Professional Vario Amperometric Detector. Calibration curves were performed using 0.4 to 100 mgL^−1^ of myo-inositol, D-pinitol, and glucose, fructose, and sucrose analytical standards. Samples with one and five months of storage were assessed to confirm the effect of storage in commercially sealed films (40 μm polypropylene bags) at room temperature (20 ± 5 °C) on the different fractions’ sugars.

### 2.4. Statistical Analysis

Free R statistical software (V 4.4.1) was utilized through the RStudio^®^ interface for the data treatment. Every experiment was replicated three times at least, depicting descriptive analysis in tables with their mean ± standard deviation. Data assumptions of normal distribution through the Shapiro–Wilk test, homoscedasticity through the Levene test, and collinearity absence through correlation matrixes were checked before applying MANOVA for physical and technological characteristics, with a confidence level of 95% for *p*-values < 0.05 being considered statistically significant, and individual ANOVA in case of H rejection, with Tukey as post hoc tests. For variables whose data distribution was not normal, even with transformation, a nonparametric Kruskal–Wallis test was performed, with Dunn tests as post hoc indicators of differences between the evaluated fractions. Graphics show average values of the evaluated variables, with error bars displaying the standard deviation (*n* = 3 replicates) and compact letters displaying differences among the fractions as a grouping variable.

## 3. Results and Discussion

The six different yampee tuber derivatives: Yampee Peeled Flour (YPF), Whole Tuber Powder (WTP), Yampee Periderm Powder (YPP), Yampee Starch Fiber (YSF), Yampee Starch Mucilage (YSM), and Lyophilized Yampee Pulp (LYP) were successfully processed, stored, and evaluated with responses being described below.

### 3.1. Resulting Technological Performance of the Fractions

#### 3.1.1. Physical Characteristics

Technological performance results are gathered in two sections, the first being the physical characteristics presented in Table 1 and the second being the technological properties of industrial interest.

**Figure 1:**
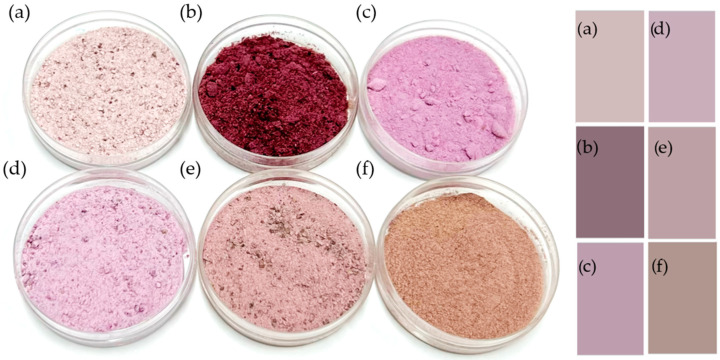
Photos of powdered fractions and simulated colors of (**a**) YSF, (**b**) YSM, (**c**) LYP, (**d**) YPF, (**e**) WTP, and (**f**) YPP.

All color parameters showed statistically significant differences (*p* < 0.05) which are clearly perceptible (Figure 1). The lightness values were higher in YPF and YSF, ranging from 72.84 ± 0.52 to 76.93 ± 0.13 units. In contrast, while WTP, YPP, and LYP had similar lightness values near a general mean, YSM exhibited the lowest one, of 48.7 ± 0.09. The a* coordinates and saturation were higher in YSM and LYP, suggesting their bioactive role as the total anthocyanin content has been correlated with this variable and, in turn, with antioxidant activity in other purple yam species flour [38]. Color space b* coordinate showed a trend in LYP, YPF, and YSM toward blue hues and in YPP, YSF, and WTP toward yellow ones.

Average chroma variations presented a tendency toward less vivid colors in YSF and WTP, followed by YPP and YPF, in comparison with more saturated YSM and LYP. LYP was the most similar to purple sweet potato flour chroma (12.66 ± 0.08) and hue (58.7 ± 0. 2), but YPF was closer to its luminosity of 72.06 ± 0.03 and YPP to its 6.581 ± 0.002 a* red hue; however, all samples differed substantially from the 10.82 ± 0.09 b* coordinate [39]. Other sources of flours with similar tones like purple maize show higher lightness around 87.5, “redness” 5.82–6.53 a* values similar to YPP and YSF, but “yellowish” b* 25.96–26.53 [40]; while purple *D. alata* yams luminosity (68.69–79.36) was similar to YPF or YSF, a* was significantly lower (2.02–4.14), and b* (4.95–11.07) was similar to YPP’s value [38]. This suggests similar uses of these products to those of purple maize or Asian yams where YSM and LYP particularly may be useful in formulations with live, red-blue hues as red velvet cakes or purplish ingredients as berry-based products, while YSF or WTP powders may be desirable for the development of foods with more neutral colors. In fact, peels of fruits such as red dragon fruit (*Hylocereus polyrhizus*) have been pureed and incorporated successfully in potato donuts, a popular food in Jakarta, with success in sensory aspects [3]. Also, natural betalain pigments in beetroot peels have replaced artificial dyes in muffins, food industry ingredients that nowadays pose a health concern [4], and, on the contrary, as dragon fruit peels in cookies, these natural pigments may add to the antioxidant capacity of foods, improving their nutritional value [5].

Water activity and moisture also exhibited differences, with the first one ranging from 0.177 (WYP) up to 0.457 (YPF), a global Aw mean of 0.303 ± 0.103, and 4.97 to 9.87 for pH in WYP and YPF, along an average moisture of 7.26 ± 2.16% across all samples. Similar values were presented in other unconventional powders such as purple *D. alata* yam flour with average moisture of 6.89–7.71% [38] or Andean tubers flour with mean water contents of 5.89–11.4% (dry basis) and 0.347–0.454 Aw [39], similar to YSM, YPF, and YSF. Other whole flours, such as taro root, present similar moisture (5.37 ± 0.13) to the one of yampee, but mucilage from the same root showed a value that is almost fourfold lower value, 2.35 ± 0.11, and the fibrous residue of 4.38 ± 0.08 [15] around half of the one found for yampee, which may be related to the fraction’s characteristics or processing conditions. The moisture level was below the recommended for flours (10–12%), meeting the requirement for all the fractions, along the water activity (<0.85), to be considered as “low-moisture foods”, traditionally known as less likely to clump, decrease in quality, or spoil, thus posing low food safety hazards because of the limited water available in them for microorganism growth under good manufacturing practices that prevent pathogenic bacteria that could survive drying [41].

Acidity ranged from 0.004 to 2.316%, while pH values were near neutrality with values from 6.44 in the lyophilized pulp to 5.6 in the periderm and the global average of 5.98 ± 0.27. This is consistent with other R&T flours, such as sweet purple potato, mashua, white, yellow, and red oca, presenting pH values of 6.1 to 7.25 [39], and cassava with pH of 4–6, including fermented roots, that show lower TSAs of 0.008–0.014%, but all, including yampee derivatives and byproducts, within safety recommendations for flours [42]. These values were also similar to the ones in whole taro root and mucilage [15] with an average pH of 5.96 to 6.67 but higher acidity than the yampee fractions. In addition to food safety, acid contents in flour or starch may affect its technological aptitude, but these characteristics are described below.

#### 3.1.2. Technological Characteristics

A correlation matrix shows coefficients among TTA and WSI (0.8502) and EA (0.8869), revealing how technological properties may be affected by product acidity. All technological properties of interest, nonetheless, show significant differences among the fractions, as presented in Table 2.

A correlation matrix among these properties linked WAI and SP with a 0.8785 coefficient, and a 0.9697 for LBD with PBD, revealing their interaction. The YPF had already proven a higher yield compared with other R&T flours, with similar luminosity, WSI, SP, and WAI to some yam, wheat, and quinoa flours, but lesser WSI, WAC, OAC, EA, and WSI to other yam flours as well as a lower acidity and higher pH, with both low moisture and Aw suggesting a long-term storability [32]. Samples of YSF showed the lowest WSI, WAI, and SP (2.49, 3.12, and 3.17, respectively). Peeled flour presented the highest LBD and PBD, with a high AD value similar to YSM, but the lowest OAC and EA (along with WTP). The periderm showed the highest WAI, SP, WHC, WAC, and OAC and the lowest LBD and AD. The whole tuber presented the lowest WAC, and mucilage showed the highest EA and WSI but the lowest PBD and WHC. It is worth noting that no extraction additives were used for the starch obtention because utilization of citric acid or sodium metabisulfite might interfere with both the yield and technological properties of the starch and its byproducts, including the yield or solubility, for example, as proved for purple sweet potato [6].

In comparison with other similar ingredients, densities of Andean tuber powders [39] between 0.57–0.67 (LBD) were close to those of yampee’s fractions with an LBD global average of 0.53 ± 0.13 and with a PBD between 0.47–0.92; they were also close to the yampee fraction mean of 0.70 ± 0.14. The packed or tapped density was slightly higher than the 0.46–0.68 PBD in *D. alata* [43] but very similar to that of extruded cassava flour 0.5–0.8 PBD [44], while the apparent density was higher than that for different varieties of cassava from the Ivory Coast of 0.49–0.62 g/mL [45]. In general, this suggests a very similar behavior of these fractions to other R&T powders or flours where PBD high values are desirable for packaging efficiency like the one in YPF and where an AD near 1.0 indicates a compact, less porous material like the one in almost all fractions but YPP.

Water solubility—from 2.36 to 27.56%—was similar in flours of different cassava varieties [45] to those of yampee fractions—of 2.28 to 26.27%—but lower than extruded flours from the cassava, ranging from 6.0 up to 56.5 [44], and lower than the 17.68–28.9% of Andean tuber flours [39]. Higher values shown by the YSM suggest this may be a useful ingredient for foods to dissolve in this kind of polar solvent like instant soups or drinks.

Other important properties, such as WAI ranging from 2.78 to 4.83, with a mean of 3.54 + 0.61, was similar to 2.6–33 in cassava [44], which, like YPP, can improve texture in baked goods and prevent dry, mealy goods. For WHC, yampee presented considerably higher values than purple maize, basul, quinoa, soy, and wheat flours [33,40], making YPP, YSF, and YPF ideal for products where a moist texture is desirable, like meat analogs or baked products. WAC was lower than those of cassava derivatives [44,45], suggesting the potential, among the evaluated fractions, of YPP for the improvement of dough handling and consistency in the first stages of preparation of these products. It is worth noting that 15.87 ± 4.28% of the total weight of the tuber was made up of peel, a material that is usually discarded for human consumption by most local farmers and artisanal processors and shows such advantages for its interaction with water. Examples of R&T peel potential usage may be found in potato peel flours, which display similar bulk density and OAC, higher WAC and SP, and almost threefold lower EA, and which have successfully enriched cakes with sensory acceptability [46]. Additionally, further processing of peel flours for the extraction of hydrocolloids has resulted in useful ingredients for home and industrial use, taking advantage of sweet potato, cocoyams (*Colocasia esculenta*), and *D. rotundata*—*D. alata* yams periderm that is otherwise wasted [47].

In terms of OAC, yampee fractions revealed a lower magnitude than that of purple yam and cassava flours [43,44,45]. However, due to their high interaction with the hydrophilic portion of water–oil mixtures, most probably, yampee fractions showed a high EA with global means of 70.008 + 12.03, ranging from 59.85 up to 96.44, which shows how yampee derivatives can be powerful emulsifying agents with magnitudes greater than that of cassava, basul, quinoa, soy, and wheat flours [33,45]. This suggests that particularly YSM may be a valuable additive in foods with both fatty and aqueous components like gravies, desserts, sauces, drinks, or the like, as with yam (*D. rotundata*) mucilage that has already proven to be an efficient emulsifier in ice cream [48], improving both the nutritional value and stability properties of the dessert. Likewise, *D. opposita* mucilage rheology studies have shown how it successfully interacts with salts, sugars, at different pH and temperatures as a thickening or gelling agent, with a considerable antioxidant activity [49]. Peels from “Spunta” potato tubers have been incorporated as powders in cakes, decreasing the baked goods’ hardness, improving color, and increasing the fiber content of the finished product with sensory acceptability, thus contributing to the functional potential of the food [7]. A similar swelling power (2.58 to 4.68 cm^−3^·g) from such tuber peels to the yampee fractions suggests the application possibilities of the fractions to modulate texture in the baking industry. Studies using lyophilized yam mucilage have already shown its competitiveness against commercial bread improvers, with acceptable texture, flavor, aroma, and a high preference for the mucilage-added breads, which also presented a lower caloric value and greater protein and carbohydrate contribution than the market additive ones [50]. With current consumer trends demanding gluten-free products and many options of the market having starch-based formulations of low bioactive content, the enrichment with red and purple-fleshed potato starch solid byproducts has provided fiber and phenolics for the coeliac-safe breads, without risking sensory, physical, or safety characteristics, proven to be acrylamide-free too [9].

### 3.2. Bioactives Found in Purple Yampee Derivatives and Byproducts

#### 3.2.1. Phytochemicals Identification

In other R&T derivatives, such as taro root, mucilage is the fraction with the highest polyphenols and anthocyanin contents, followed by whole flour, and starch-derived fibrous residue [15]. Preliminary experiments revealed the detectable highest amounts for LYP and YSM yampee fractions. Then, HPLC-QToF determinations focused on these two powdered fractions, and twenty phytochemicals were identified (Table 3). The compounds of interest were organic acids, nucleosides, hormone derivatives, flavonoids, and other phenolic compounds. Edible ingredients from acidified water extracts of *D. trifida* have recently reported the presence of quinic acid and rhamnetin derivates such as isorhamnetin-O-dihexoside, similarly to what was found in this work on purple yampee species grown in South America [10]. In Jamaican *D. alata* tubers, azelaic acid was reported, such as found in yampee, and authors highlight the antioxidant power of the aqueous extracts, and their anticancer activity [11], which may suggest a potential benefit of yampee extracts yet to be studied for their therapeutical use.

In other *Dioscorea* yams as *D. polystachya*, compounds such as allantoin and citric acid have been identified in both the tubers’ cortex and/or flesh [12]. Malic acid is also present in *D. elephantipes* [13], a species in which the authors highlight, as is also the aim of this study, the wide array of renewable sources for metabolites in biodiversity, with yams being an example of some high-value phytochemicals underutilized to the day. As our work also promotes efficient comprehensive use of edible materials, it is also worth noting that not only tubers but leaves of *Dioscorea* species have been found to bear an assortment of bioactive compounds, including sugars, sugar alcohols, fatty acids, amides, allantoin, uridine, adenosine, amines, and amino and organic acids [15], that are also a neglected source of substances for nutraceutical, food, or even pharmaceutical use.

It is worth noting how processing may affect bioactive compounds in *Dioscorea* species as many of these compounds are highly labile when exposed to extreme temperatures, extensive mechanical damage, or radiation. Additionally, scalable methods for smallholders often introduce one or more of these abiotic stresses. Although methods used to obtain different fractions from yampee strived to decrease such unfavorable circumstances, it is been noted how flour obtention conditions may affect prebiotic compounds such as inulin or antioxidant citric acid, which is the second most abundant organic acid in *D. esculenta* and *D. bulbifera* yams flour [14]. The difference in the presence of bioactives (including anthocyanins, amino acids, and others) in yampee fractions could be attributed not only to their nature but to those conditions, and the results from this research should be used as a foundation for future optimization of such techniques to preserve and maximize phytochemical harnessing. One of the indicators of such stress in the fractions may be the identification of jasmonate derivatives in the samples as this is a compound widely renowned for its role in the reaction to different types of distress, triggering physiological returns that may range from tissue decay to anthocyanin biosynthesis [16]. Endogenous jasmonates and cytokinins are accumulated during tuber enlargement, as in potatoes, playing a vital role in the tubers’ formation and growth, declining toward senescence, and improving tuberization in *D. alata*, *D. batatas*, *D. rotundata*, and *D. cayenensis* [17]. As a modulator on postharvest physiological behavior, the presence of jasmonate-related compounds in yampee fractions also suggests their use not only as an edible additive but also as a GRAS ingredient to use in the preservation of other fresh products, including tubers whose dormancy may be prolonged, fruits whose ripening may be delayed, or bioactives such as the abovementioned anthocyanins, whose content may be increased for both sensory or functional purposes.

#### 3.2.2. Amino Acids Identification and Quantification

**Figure 2:**
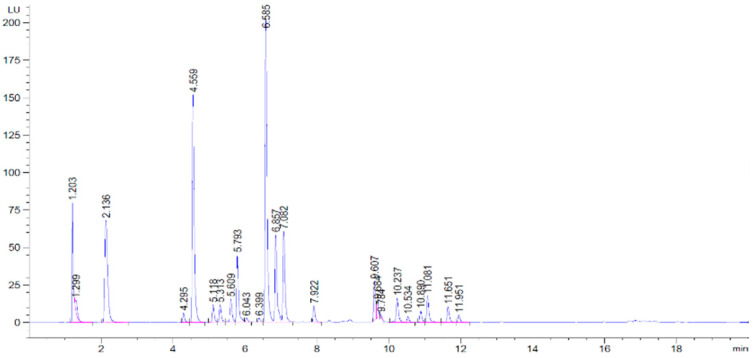

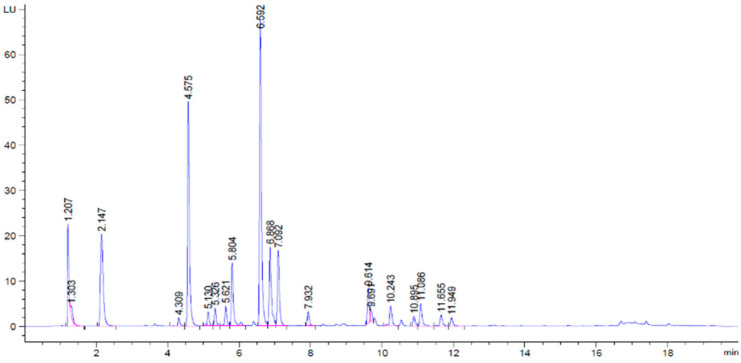
Amino acids in *D. trifida* mucilage (**upper** chromatogram) and lyophilized pulp (**under**) observed through HPLC-FLD.

Determination of amino acids using chromatographic techniques and fluorescence detection resulted in notable amounts in yampee mucilage and lyophilized pulp fractions. The main amino acidic peaks detected are shown in Figure 2, with corresponding identification and amounts depicted in Table 4.

The twelve amino acids found in the samples included essential isoleucine, leucine, lysine, threonine, valine, and “conditionally” essential histidine and arginine. Contents of amino acids were in general greater in the mucilage fraction, probably because of an increase in the molecule’s bioavailability due to the absence of the predominant starchy element in the dried tuber, which may sterically impede the peptide detection. Another possible explanation for greater levels of amino acids in the mucilage is the presence of glycoproteins bound to the carbohydrate part removed during starch extraction, which may leave on its way an effective chelating antioxidant as other mucilages like taro [18] has proven to be. The main amino acids contained in the samples were arginine, followed by aspartate and serine, which may not be essential, but have a great role in human health promotion as they are involved in protein synthesis, immune function, growth, energy production, neurotransmission, fats metabolism, or brain function among others [19].

Samples of other yams, such as *Dioscorea alata*, *D. bulbifera* var. vera, *D. esculenta*, *D. oppositifolia* var. oppositifolia, *D. oppositifolia* var. dukhumensis, *D. pentaphylla* var. pentaphylla, *D. spicata*, *D. tomentosa*, and *D. wallichi*, have also proven to bear these amino acids in variable concentrations in comparison with the yampee [20]. Although a genetic variation intrinsic to the species and varieties is given, nutrition during cultivation, times of drought, or excessive watering when hard climate strikes may be among the elements influencing the tubers’ overall quality and, also, storage and processing [21] as postharvest implies a nutrient exchange to ensure the tuber survival, as well as preparation toward dormancy break, with demands for building new tissues and active metabolism for new growth.

Additionally, studies focusing on recovering proteins from wastewater mucilage from *D. opposita* have found that its composition is dominated by dioscorin isoforms rich in glutamic and aspartic acid, with the presence of essential amino acids also detected. The bioactive character of the protein’s radical scavenging is related to the proton donation ability from the imidazole group in histidine, the hydrogen donation of tyrosine or phenylalanine phenolic groups, or methionine oxidation [22]. Yampee mucilage also showed the presence of histidine and tyrosine, suggesting its role as an antioxidant ingredient. This bioactivity has already been reported in yampee products such as tuber flour and its derivatives [26,27].

#### 3.2.3. Anthocyanin Identification and Quantification

The exploration of flavonoid pigments through HPLC-ESI-QTOF-MS resulted in the identification of twelve compounds (Table 5), corresponding to anthocyanidin pigments in the samples after 20-day storage from their manufacture.

In comparison with previous reports of *D. trifida* [22], similar anthocyanins were identified, with a slightly lower intensity of the peaks. This fact is probably related to the transportation of the samples to the analysis site, involving storage time and handling, in addition to the samples’ processing, probably with a deleterious effect on these labile compounds. The main anthocyanins in the common edible purple yam, *D. alata* include cyanidin, pelargonidin, peonidin, and alatanins [51]. In this case, in agreement with studies on Peruvian samples of *D. trifida* [22], main pigment found for Colombian *D. trifida* was peonidin 3-*O-p*-cumaroylglucoside-5-*O*-glucoside and no malvidins were registered in any of the fractions, in contrast to what was documented in Venezuelan accessions [52], which reported the presence of a di-glucoside of the anthocyanidin and a ferulic acid derivative as well. Due to the steric hindrance of acids against water interactions, the formation of chalcones or achromatic pseudobases, in addition to the barrier against breakdown and their oxidation preventing oxygen effect on anthocyanins, *p*-coumaric acid was shown to add stability to the pigments [53]. Depending on the degree of acylation, and the specific acid bound to the anthocyanin, stability may be increased as caffeic acid has demonstrated greater stability in comparison with ferulic in purple sweet potato [54]. In contrast to previous findings on the species, new reports of petunidin derivatives were found, along with other pelargonidin ones, suggesting a greater diversity of phytochemicals in the tubers grown in the Colombian Caribbean, which may even be greater with a shorter time of analysis (rather fresh samples) or different processing techniques from the raw material and could be of interest for bioactive extraction. Petunidins have been previously reported as responsible for the “deep-purple color” in the tuber skin and flesh, with a tissue and genotype-specific pattern for the pigment’s composition, but contributing majorly to the total anthocyanin content (63–66%), led by petunidin-3-*p*-coumaroylrutinoside-5-glucoside and petunidin-2-*p*-coumaroylrutinoside-5-glucoside [25]. In comparison to other tubers, although acylated glycosides of anthocyanidins are also present in underground storage organs of purple color, in potatoes, malvidin, delphinidin, petunidin, and peonidin are the main type of pigments, while a red hue of the tubers is related to those of acylated glycosides of pelargonidin, with their corresponding transcriptome profiles [55], yet to be explored for purple yampee. Interestingly, although phylogenetically distant as well, yampee shares cyanidin and peonidin as main pigments with sweet potatoes—another member of the Solanales order, closer to potatoes—also acylated with ferulic acid [56] as the peonidin found in this study.

**Figure 3:**
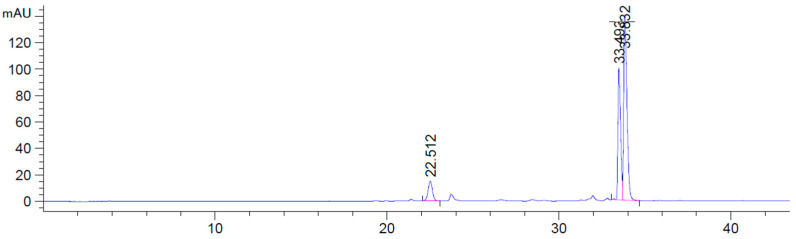
Main anthocyanin compounds identified at 520 nm through HPLC-DAD in *D. trifida* fractions after 20 days from their manufacture.

Among the identified anthocyanins (Figure 3), three predominant peaks (number 1, 7, and 9 in Table 3) were reported, which was consistent with results in *D. trifida* lyophilized samples [57]. These were further quantified through HPLC-DAD by interpolating with the peonidin standard curve (R^2^ = 1), which was also used for equivalent determination.

Peonidins were the main anthocyanins found in all samples, with the most abundant being Peonidin 3-O-*p*-cumaroylglucoside-5-O-glucoside, followed by the Peonidin 3-O-feruloylglucoside-5-O-glucoside, and Peonidin 3-O-5-Glucoside, respectively (Figure 3).

**Figure 4:**
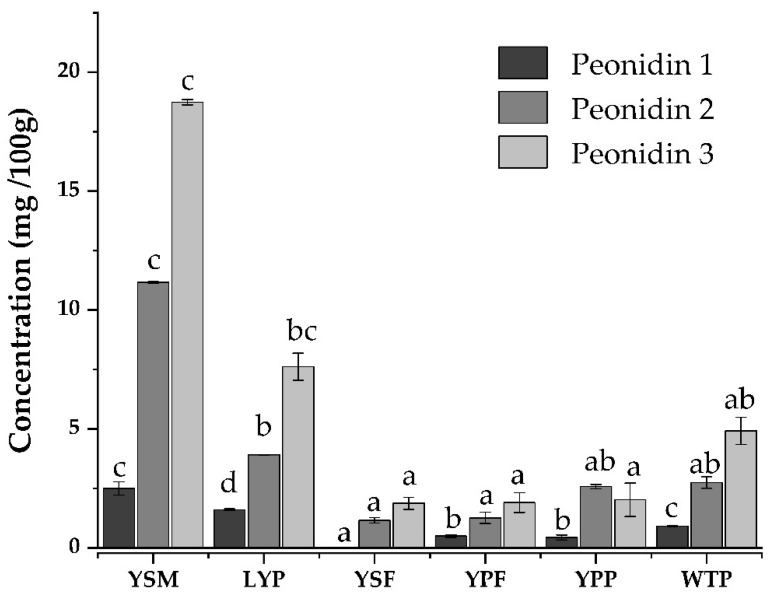
Main anthocyanins quantified in *D. trifida* fractions mucilage YSM, lyophilized pulp LYP, starch fiber, flour, periderm, and whole flour from left to right, after 20-day storage. Main peonidin derivatives are depicted as follows: Peonidin 3-*O*-glucoside-5-*O*glucoside (Peonidin 1), Peonidin 3-O-feruloylglucoside-5-O-glucoside (Peonidin 2), and Peonidin 3-O-*p*-cumaroylglucoside-5-O-glucoside (Peonidin 3). The average concentration of the molecules (*n* = 3) is presented in milligrams per 100 g of sample, letters correspond to significant statistical differences among fractions (*p* < 0.05).

As shown in Figure 4, lower amounts of peonidins were detected in YSF, YPF, and YPP (from 1.32 to 1.63 mg of “Peonidin 3” per 100 g of sample), intermediate values in WTP (4.92 ± 0.57 mg of “Peonidin 3” per 100 g of sample), and higher in YSM and LYP (up to 8.18 and 18.85 mg of “Peonidin 3” per 100 g of sample, respectively).

As complementary fractions, low values in YPF might be explained by higher amounts in YSM, with other functional compounds possibly bound to different molecules in fiber structure. Low values in peels were also expected as purple flesh usually concentrates pigments, and periderm has been reported to be rich in other phenolic and carbohydrate-like compounds [46,47,58,59]. Thermal processing is recognized to change anthocyanin color because of ring-opening degradation and hydroxyl groups conferring instability to the pigments [60]; therefore, conventional cabinet drying performed on exposed yampee flesh in YPF was also expected to be in lower amounts. In these terms, preserving periderm in WTP may well add itself to the overall anthocyanin amount in the fraction or act to protect the pigments to some extent, or the mere absence of processing and overhandling of YPF, with the light, temperature, and oxygen exposure that manual peeling exerts on the tuber, may be responsible for these ingredients approximately doubling the peonidin content in the whole tuber. Compared with other determinations, 20 days after the production and transportation, mucilage showed similar amounts of Peonidin 3-*O*-glucoside-5-*O*-glucoside (2.39 mg 100 g^−1^), higher amounts of Peonidin 3-O-feruloylglucoside-5-O-glucoside (4.43 mg 100 g^−1^) as freeze-dried pulp, and lower amounts of Peonidin 3-O-*p*-cumaroylglucoside-5-O-glucoside (22.92 mg 100 g^−1^) than lyophilized samples of Brazilian *D. trifida* accessions [57].

As anthocyanin amounts were higher in YSM and LYP, their evolution after a four-month storage period, sealed at room temperature (20 °C) in darkness was recorded. YSM samples showed a 36% decrease in Peonidin 3-O-*p*-cumaroylglucoside-5-O-glucoside and a 43% in the other two peonidins, while LYP diminished its Pn 3-O-5-G by 34%, its Pn-3-O-ferG-5-O-G by 47%, and its Pn-3-O-*p*-cuG-5-O-G average content by 48%. This highlights that, in addition to the technological advantages of the YSM, this fraction offers a greater overall stability in its bioactive potential.

#### 3.2.4. Sugars Quantification and Evolution

**Figure 5:**
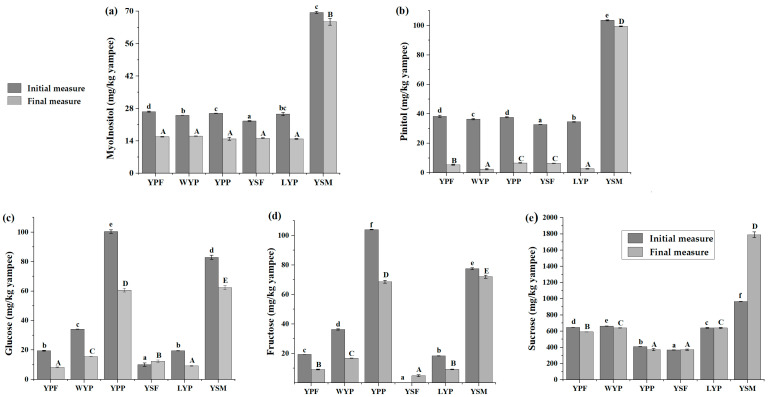
The evolution of sugars in time (day 43 -darker color- and 180 of storage) for each fraction depicted by individually quantified sugars (**a**) Myo-inositol, (**b**) Pinitol, (**c**) Glucose, (**d**) Fructose, (**e**) Sucrose. Concentrations of each measure are depicted as mean values with standard deviation error bars (*n* = 3). Means in a column followed by different lowercase letters (a–f) are significantly different at the 5% level at the initial time of measure. Means in the columns followed by different capital letters (A–E) are significantly different at the 5% level.

All evaluated sugars were found in the six fractions of the purple yampee (Figure 5), with a significant change for some of them in different fractions.

Mucilage was the fraction with the highest amounts of Myo-Inositol, D-Pinitol, and sucrose (69.50 ± 0.46, 103.54 ± 0.34, and 1788. 88 ± 34. 96 ppm, respectively), the one to preserve the greater amount of glucose and fructose. Sucrose levels were also higher in YSM in comparison with all fractions, standing out along YPP in glucose contents both freshly extracted and stored for a few months. Fructose concentrations presented fewer variations, particularly after storage of the samples, with higher values for YPP and YSM and lower values for YSF and LYP.

Sucrose, glucose, and fructose are a readily available source of energy useful for athletes. They industrially act as sweetening, water-retaining agents, and reducing sugars for Maillard reactions during baking, improving sensory characteristics of baked products, in addition to being carbon sources during fermentation and serving as preservatives by reducing Aw in the case of glucose. Inositol polyols, such as myo-inositol—renowned as the most abundant in nature—and methylated D-pinitol—most common plant-derived inositol—in addition to a technological role as most sugars, are involved in metabolic homeostasis, cell growth, osteogenesis, apoptosis, stress response, reproductive functions, and neural and fetal development, with antidiabetic, antioxidant, anticancer and chemopreventive activities [61,62].

According to the report on the sugars in more than 40 plants, including roots, leaves, and other vegetable organs, myo-inositol concentration was lower than turmeric roots (0.1489 mg g^−1^), dandelion root (0.1243 mg g^−1^), carrot (0.8001 mg g^−1^), similar to parsley roots (0.0329 mg g^−1^) and higher than ginger roots (0.0087 mg g^−1^), and potato (0.0141 mg g^−1^), among different plants described [63]. The remaining sugars evaluated were lower than those of carrots, ginger, parsley roots, and dandelion roots. This may suggest an advantage for the fractions’ composition as they may constitute functional ingredients with a lower glycemic peak when consumed but increased bioactive potential by containing the aforementioned polyols that have been demonstrated to prevent mental health conditions and even metabolic syndrome [64,65].

Underground storage organs, tubers, and, therefore, most of their derivatives, are rich in polysaccharides. *D. opposita* yams have exhibited a pseudoplastic “gel-like” behavior whose viscosity-modulation power can be modified with high temperatures, extreme alkaline, or acid conditions [29], which indicates yams’ carbohydrate potential for use in the processing of soups, beverages, or deserts in a wide range of pH and thermal fluctuations. In addition to being a very important ingredient in food design, polysaccharides have also been distinguished for having a crucial structural role in edible coatings as environmentally friendlier alternatives to other polymers, and ginseng root byproducts, rich in polysaccharides, have successfully preserved strawberries and fresh-cut apples [30]. Incorporation of yam fractions could be a great possibility in this field as *D. opposita* polysaccharide-rich mucilage (45% *w*/*w*) in edible films has served as an emulsifier, as well as an oxygen-blocking agent for its polymeric well-ordered hydrogen bond network, with the advantage of nontoxicity, biodegradability, low cost, and procuring effort [66].

Collectively the results of these fractions serve the optimization of the resources for economic benefit, as well as environmental pressing issues related to circular economy, by avoiding inefficient disposition of byproducts and, ultimately, improving social conditions of farmers who will not deal with contaminated water bodies, decomposing organic matter, or the mere resource loss, adding to the production increase demand already latent.

## 4. Conclusions

This study highlights the potential of yampee as a sustainable and valuable source of bioactive compounds, particularly anthocyanins and other phenolic compounds known for their remarkable antioxidant properties. Our findings suggest that the freeze-dried mucilage and pulp, with their high color saturation and elevated anthocyanin content, are ideal for developing colored foods with enhanced nutritional value. The periderm and mucilage fractions demonstrated significant suitability for use as additives in meat analogs and baked goods as their water-holding capacity (WHC), water absorption capacity (WAC), water solubility index (WSI), and emulsifying activity (EA) can enhance the palatability of these products. Additionally, the mucilage showed the highest anthocyanin and functional sugar content, such as pinitol and myo-inositol, with less degradation over a 4-month storage period. These findings underscore the considerable nutritional, technological, and biological potential of industrial process residues, promoting the development of functional foods from a circular economy perspective.

## Figures and Tables

**Table 1:** Physical characteristics of the yampee-derived fractions (presented as mean ± SD, *n* = 3).

	YPF	WTP	YPP	YSF	YSM	LYP
L*	72.84 ± 0.52 ^d^	67.71 ± 0.49 ^c^	63.25 ± 8.69 ^b^	76.93 ± 0.13 ^e^	48.7 ± 0.09 ^a^	67.01 ± 0.10 ^c^
a*	8.69 ± 0.13 ^c^	7.72 ± 0.16 ^b^	6.57 ± 0.17 ^a^	6.31 ± 0.07 ^a^	10.34 ± 0.00 ^d^	10.33 ± 0.05 ^d^
b*	−3.02 ± 0.02 ^b^	1.9 ± 0.16 ^d^	6.61 ± 0.14 ^f^	2.46 ± 0.08 ^e^	−1.46 ± 0.05 ^c^	−4.65 ± 0.03 ^a^
C*	9.19 ± 0.12 ^c^	7.95 ± 0.19 ^b^	9.32 ± 0.21 ^c^	6.78 ± 0.09 ^a^	10.44 ± 0.00 ^d^	11.32 ± 0.06 ^e^
h*	19.13 ± 0.44 ^c^	13.81 ± 0.90 ^b^	45.15 ± 0.51 ^f^	21.33 ± 0.46 ^d^	8.00 ± 0.28 ^a^	24.20 ± 0.12 ^e^
Aw	0.451 ± 0.004 ^c^	0.194 ± 0.015 ^a^	0.229 ± 0.009 ^a^	0.420 ± 0.002 ^c^	0.299 ± 0.037 ^b^	0.223 ± 0.011 ^a^
Moist (%)	9.59 ± 0.39 ^c^	4.99 ± 0.03 ^a^	5.35 ± 0.14 ^a^	9.74 ± 0.12 ^c^	8.65 ± 0.11 ^b^	5.22 ± 0.03 ^a^
pH	5.75 ± 0.01 ^a^	6.08 ± 0.01 ^b^	5.69 ± 0.13 ^a^	5.90 ± 0.39 ^ab^	6.07 ± 0.01 ^b^	6.43 ± 0.02 ^c^
TTA (%)	0.362 ± 0.006 ^a^	0.177 ± 0.047 ^a^	0.709 ± 0.049 ^a^	0.359 ± 0.007 ^a^	1.820 ± 0.495 ^b^	0.265 ± 0.227 ^a^

Color is expressed in CIELab* space coordinates for lightness (L*), a* and b* coordinates, as chroma (C*) and hue (h*) for corresponding values of these attributes. Total titratable acidity (TTA) results are given in % of the predominant acid (succinic) in the sample and moisture (moist) as the percentage of water in the sample. Different letters indicate statistical significance (*p* ≤ 0.05).

**Table 2:** Technological properties of the yampee fraction mean values ± SD (*n* = 3).

	YPF	WTP	YPP	YSF	YSM	LYP
LBD *	0.72 ± 0.04 ^c^	0.56 ± 0 ^b^	0.37 ± 0.01 ^a^	0.60 ± 0.01 ^b^	0.39 ± 0 ^a^	0.56 ± 0 ^b^
PBD	0.90 ± 0.02 ^e^	0.73 ± 0.02 ^c^	0.56 ± 0.02 ^b^	0.79 ± 0.02 ^d^	0.48 ± 0.01 ^a^	0.75 ± 0.02 ^cd^
AD	0.81 ± 0.03 ^b^	0.77 ± 0.03 ^b^	0.67 ± 0.02 ^a^	0.76 ± 0.00 ^b^	0.81 ± 0.00 ^b^	0.75 ± 0.02 ^b^
WSI	3.80 ± 0.08 ^a^	4.25 ± 0.11 ^a^	5.18 ± 0.13 ^a^	2.49 ± 0.29 ^a^	22.4 ± 5.46 ^b^	4.29 ± 0.08 ^a^
WAI	3.19 ± 0.12 ^a^	3.32 ± 0.08 ^a^	4.72 ± 0.1 ^b^	3.12 ± 0.12 ^a^	3.49 ± 0.63 ^a^	3.39 ± 0.25 ^a^
SP	3.27 ± 0.17 ^a^	3.42 ± 0.02 ^a^	4.89 ± 0.19 ^b^	3.17 ± 0.11 ^a^	4.14 ± 0.8 ^ab^	3.49 ± 0.17 ^a^
WHC	8.11 ± 0.02 ^c^	6.47 ± 1.21 ^b^	10.02 ± 0.08 ^d^	8.63 ± 1.04 ^c^	2.92 ± 0.55 ^a^	7.45 ± 0.21 ^bc^
WAC	1.86 ± 0.05 ^b^	1.59 ± 0.07 ^a^	3.30 ± 0.03 ^e^	2.21 ± 0.03 ^d^	1.99 ± 0.18 ^bc^	2.12 ± 0 ^cd^
OAC	0.86 ± 0.03 ^a^	1.09 ± 0.07 ^b^	1.85 ± 0.12 ^d^	1.07 ± 0.06 ^b^	1.57 ± 0.02 ^a^	1.10 ± 0.15 ^b^
EA	62.27 ± 3.41 ^a^	62.34 ± 1.5 ^a^	66.38 ± 4.08 ^ab^	64.86 ± 3.03 ^ab^	95.42 ± 1.43 ^c^	68.77 ± 1.33 ^b^

* Loose bulk density (LBD) and packed bulk density (PBD) are given in gml^−1^; emulsion activity (EA), water solubility index (WSI) in %, units; water holding capacity (WHC) in grams of water per grams of flour; water absorption capacity (WAC) in absorbed water (g) per gram of sample; oil absorption capacity (OAC) in grams of absorbed oil per gram of sample; while the rest of variables are dimensionless. Different letters indicate significant statistical differences (*p* ≤ 0.05).

**Table 3:** Major tentative compounds identified in methanol/water (1:1) extracts of *D. trifida* obtained by HPLC-ESI-QTOF-MS.

Peak	Rt (min)	Tentative Assignment	CAS Number	Adduct Type	Observed *m*/*z*	Main MS/MS Fragment Ions (*m*/*z*)	
1	3.064	Allantoin	97-59-6	[M + H]^-^	157.0354	114.0296/96.9588/71.0125/59.0143	[12]
2	3.211	Quinic acid	77-95-2	[M + H]^-^	191.058	173.0473/127.0413/93.0354/85.0303/59.0144	[10]
3	3.887	Malic acid	6915-15-7	[M + H]^-^	133.0155	115.0049/89.0253/72.0939/71.045/59.0143	[13]
4	4.385	Isocitrate	320-77-4	[M + H]^-^	191.0199	173.0091/154.9986/111.0087/101.02442/85.029/73.0295	*
5	7.723	Citric acid	77-92-9	[M + H]^-^	191.022	129.0195/111.009/87.0085/85.0295/67.0184/57.0340	[12]
6	9.035	Citric acid derivate	N/A	[M + H]^-^	-	191.0193/129.0192/87.0092/85.0298/59.0142/57.0349	*
7	9.072	Succinic acid	110-15-6	[M + H]^-^	117.0187	101.0238/99.0082/73.0287/55.0185	[14]
8	9.772	Citric acid derivate	N/A	[M + H]^-^	-	191.0196/129.0195/111.0089/87.0089/67.0197	*
9	13.33	Adenosine	58-61-7	[M + H]^-^	266.0894	134.0459/107.0363	[12]
10	17.345	Glucogallic acid	84274-52-2	[M + H]^-^	331.0685	164.0718	[12]
11	29.338	Isopropylmalic acid	3237-44-3	[M + H]^-^	175.0618	157.0509/131.0723/113.0611/ 101.0242/85.0663/72.9931/69.0706	*
12	33.666	Benzoquinoneacetic acid	10275-07-7	[M + H]^-^	166.0267	121.0298/140.0474/93.0346/77.0395	*
13	36.14	Kaempferide derivative	N/A	[M + H]^-^	623.1601	299.0556	[15]
14	36.82	2-O-Feruloylhydroxycitric acid	62345-86-2	[M + H]^-^	383.0622	189.0040/134.0373/59.0138	*
15	38.1	Rhamnetin 3-galactoside- 4′-glucoside	N/A	[M + H]^-^	639.1577	315.0572	*
16	38.494	Epigallocatechin 7-glucuronide	569670-42-4	[M + H]^-^ + [H_2_O]	463.0897/ 482.1075	169.043/125.0244/57.0345	*
17	39.338	Rhamnocitrin-3-(5″-ferulylapiosyl)-(1 → 2)glucoside	148210-00-8	[M + H]^-^	769.1987	299.0552/178.026	*
18	40.589	Azelaic acid	123-99-9	[M + H]^-^	187.0978	169.087/143.1077/125.0973/97.0659/57.0345	[11]
19	47.185	Dihidrojasmonic acid derivative	N/A	[M + H]^-^	-	211.1338/167.1445/59.0135	*
20	50.194	Dihidrojasmonic acid derivative	N/A	[M + H]^-^	-	211.1340/167.1441/59.0142	*

Rt, retention time. Identification was conducted using METLIN library version 8.0 and MS fragmentation patterns described previously by cited studies on the right column. Compounds with an * were identified through the aforementioned compound database.

**Table 4:** Identification and concentration of amino acids in yampee mucilage and pulp (mg of amino acid per 100 g of sample, mean ± SD, *n* = 3). * Samples portraying < LOQ refer to values below the limit of quantification (LOQ).

Peak	Rt (min)	Fraction	Pulp	Mucilage
1	1.203	Aspartate	101.91 ± 7.71	733.17 ± 72.99
2	4.559	Serine	101.45 ± 9.02	504.33 ± 28.03
3	5.313	Histidine	<LOQ *	103.14 ± 5.63
4	5.609	Glycine	<LOQ	38.71 ± 3.05
5	5.793	Threonine	30.5 ± 3.93	261.57 ± 11.97
6	6.585	Arginine	230.14 ± 10.85	1352.75 ± 35.19
7	6.857	Alanine	30.82 ± 4.16	217.75 ± 10.4
8	7.082	Tyrosine	59.78 ± 2.34	460.44 ± 34.77
9	9.607	Valine	17.26 ± 1.59	112.39 ± 10.94
10	11.081	Isoleucine	<LOQ	145.82 ± 9.01
11	11.651	Leucine	<LOQ	52.69 ± 9.32
12	11.951	Lysine	33.84 ± 4.15	138.4 ± 19.49

**Table 5:** Mass spectral data and tentative identification of anthocyanins in *D. trifida* fractions obtained by HPLC-ESI-QTOF-MS.

Peak	Rt (min)	[M]^+^ (*m*/*z*)	Main MS^2^ Fragment Ions (*m*/*z*)	Tentative Identification
1	19.279	625.1788	301.0726	Peonidin-3-O-glucoside-5-O-glucoside *
2	21.818	595.1696	271.0616	Pelargonidin derivative
3	24.13	787.2132	287.0570	Cyanidin-3-O-feruloylglucoside-5-O-glucoside *
4	24.923	757.20132	287.0567	Cyanidin-3-O-p-coumaroylglucoside-5-O-glucoside *
5	25.421	561.1589	271.0618	Pelargonidin derivative
6	26.366	831.2389	301.0728	Peonidin derivative
7	27.986	771.2176	271.0619	Pelargonidin derivative
8	28.613	801.2299	301.0733	Peonidin 3-O-feruloylglucoside-5-O-glucoside *
9	29.176	741.2067	271.0619	Pelargonidin-3-O-p-coumaroylglucoside-5-O-glucoside *
10	30.031	771.2187	301.0734	Peonidin 3-O-p-cumaroylglucoside-5-O-glucoside *
11	36.288	641.1754	317.0674	Petunidin derivative
12	38.963	625.1796	317.0678	Petunidin derivative

* These compounds were identified in agreement with previous reports of *D. trifida* anthocyanins from Peruvian accessions [28]. MS^2^ refers to the resulting secondary fragments detected in addition to the precursor ion [M]^+^ for each compound.

## Data Availability

Collected data is available for public consultation on Zenodo UPCT community through Foods3346197 link.
